# Anticoagulation in non-malignant portal vein thrombosis is safe and improves hepatic function

**DOI:** 10.1007/s00508-018-1351-y

**Published:** 2018-06-18

**Authors:** Bernhard Scheiner, Paul René Stammet, Sebastian Pokorny, Theresa Bucsics, Philipp Schwabl, Andrea Brichta, Johannes Thaler, Katharina Lampichler, Ahmed Ba﻿-﻿Ssalamah, Cihan Ay, Arnulf Ferlitsch, Michael Trauner, Mattias Mandorfer, Thomas Reiberger

**Affiliations:** 1 Vienna Hepatic Hemodynamic Lab, Division of Gastroenterology and Hepatology, Department of Internal Medicine III, Medical University of Vienna, Waehringer Guertel 18–20, 1090 Vienna, Austria; 2 Vienna Hepatic Hemodynamic Laboratory, Medical University of Vienna, Vienna, Austria; 3 Clinical Division of Hematology and Hemostaseology, Department of Medicine I, Medical University of Vienna, Vienna, Austria; 4 Department of Radiology, Medical University of Vienna, Vienna, Austria

**Keywords:** Liver diseases, Portal vein, Venous thrombosis, Anticoagulants

## Abstract

**Background:**

Non-malignant portal vein thrombosis (PVT) is common in patients with advanced liver disease. Anticoagulation (AC) increases the chances of recanalization and may improve liver function in patients with cirrhosis.

**Aim:**

We retrospectively assessed the course of non-malignant PVT in patients receiving AC.

**Methods:**

Parameters related to hepatic injury (aspartate aminotransferase [AST]/alanine aminotransferase [ALT]), severity of disease (ascites) and synthesis function (albumin) as well as AC, rates of PVT regression/progression and AC-associated complications were documented.

**Results:**

Among 122 patients with PVT, 51 patients with non-malignant PVT (27 incomplete, 24 complete) were included, 12 patients (25%) received long-term AC therapy (≥9 months) as compared to 36 patients without long-term AC. We observed a trend towards higher regression rates with long-term AC of 58% (vs. 28% without AC; *p* = 0.08) and lower progression rates of 25% (vs. 42% without AC; *p* = 0.15). In the subgroup of patients with decompensation prior to PVT diagnosis (*n* = 39), long-term AC (*n* = 10, 25.6%) resulted in a significantly higher rate of PVT regression/resolution (70% vs. 24%, *p* = 0.031). Interestingly, AST/ALT tended to decrease (−19%/−16%) and the proportion of patients with ascites became lower (−33%) with long-term AC (without AC: ±0%). Furthermore, there was a significant improvement in albumin levels (+9%/+3.6 g/dl) when compared to patients without long-term AC (−2%/−0.8 g/dl; *p* = 0.04). Additionally, 10 patients were treated with direct oral anticoagulants (DOACs) for splanchnic vein thrombosis. Importantly, there were no AC-associated bleeding events in patients with conventional AC and one bleeding event in patients with DOAC treatment (10%).

**Conclusion:**

Our findings support anticoagulation in patients with non-malignant PVT, since AC seems safe and associated with superior PVT regression rates and might also decrease hepatic injury and improve liver synthesis.

## Study highlights

- Long-term anticoagulation (AC) in patients with portal vein thrombosis is recommended by international guidelines to improve recanalization rates.
- In this study, long-term AC was associated with a trend towards higher portal vein thrombosis (PVT) regression rates, which attained statistical significance in patients with decompensated cirrhosis.
- Furthermore, long-term AC was associated with significant improvements in hepatic synthesis function (albumin).
- In patients with liver cirrhosis AC appears to be safe, as no AC-associated bleeding events occurred in patients with conventional AC and only one bleeding event in DOAC patients.

## Introduction

Cirrhosis can develop from virtually all forms of chronic liver disease with alcoholic liver disease and chronic viral hepatitis being the most common etiologies in developed countries [1]. Patients with cirrhosis are at increased risk for gastrointestinal bleeding, mainly from esophageal varices or portal hypertensive gastropathy [2, 3]. In contrast to the dogma of auto-anticoagulation in cirrhosis, venous thrombosis is more common in patients with cirrhosis than in the general population [4, 5]. There is a 7.3-fold increased risk of developing portal vein thrombosis (PVT) with cirrhosis. This represents a severe complication in patients with liver cirrhosis that may lead to intestinal infarction and preclude the option for orthotopic liver transplantation [6, 7]. The prevalence of PVT is up to 23.3% in liver transplantation candidates without hepatocellular carcinoma (HCC) and the yearly incidence of non-malignant PVT is estimated to be between 7–11% in patients with cirrhosis.

Although partial PVT in patients with cirrhosis may resolve spontaneously [8, 9], the majority of patients (48–70%) show progression of PVT within 2 years [8, 10]. Thus, anticoagulation with low molecular weight heparin (LMWH) or vitamin K antagonists (VKA) has been used for treatment of cirrhotic PVT with reported recanalization rates of 55–75% [10–14] when given for about 6 months. Since PVT might recur in up to 38% of cirrhotic patients when anticoagulation is stopped early after PVT resolution [9, 13], long-term anticoagulation has been suggested by recent European Association for the Study of the Liver (EASL) guidelines [15], particularly in liver transplantation candidates.

Since PVT can cause portal hypertension-related bleeding and anticoagulation may further increase the risk of bleeding events, the safety of anticoagulation therapy needs to be specifically addressed in patients with cirrhosis; however, in previous studies the incidence and severity of bleeding events related to anticoagulation with LMWH or VKA was low [10–14].

Next to the efficacy of anticoagulation to prevent and resolve PVT, there are experimental data supporting a beneficial impact on liver synthesis function and fibrosis [16]. In a prospective clinical trial, the administration of prophylactic LMWH did not only prevent PVT in liver transplantation candidates but also decreased hepatic decompensation and improved survival [17].

Thus, this study aimed to assess (i) regression rates of non-malignant PVT with and without anticoagulation and (ii) the evolution of hepatic inflammation and liver function in patients with PVT. Furthermore (iii) we provide a summary of the existing literature on this topic.

## Patients and methods

### Study design and inclusion of subjects

This study was a retrospective analysis on the efficacy of anticoagulation for regression of PVT and its impact on hepatic inflammation and liver function. The specific inclusion criteria for this study were patients aged ≥18 years with a diagnosis of cirrhosis and concomitant PVT. We excluded patients with malignant PVT and patients without sufficient clinical information at the time of PVT diagnosis and on the clinical course thereafter. Furthermore, data on direct oral anticoagulants (DOACs) used in a small cohort of patients for treatment of splanchnic vein thrombosis are reported.

### Clinical and radiological parameters

Clinical data was collected from patients’ medical records. Radiological images by computed tomography (CT), magnetic resonance imaging (MRI) and sonography were reviewed by a second expert radiologist in case PVT diagnosis or extension of thrombus was unclear. Demographic characteristics and comorbidities were recorded at PVT diagnosis. In addition, we longitudinally assessed the use of anticoagulation therapy with LMWH or phenprocoumon. The course of PVT was recorded as (i) regression, (ii) stable or (iii) progression. Ascites and hepatic encephalopathy were graded according to the Child-Pugh score at PVT diagnosis and at follow-up. Moreover, bleeding events during follow-up were recorded.

### Laboratory parameters

Serum levels of creatinine, bilirubin, albumin, and liver enzymes were recorded at PVT diagnosis and at follow-up. Furthermore, prothrombin time and International Normalized Ratio (INR) were assessed at both time points (baseline and follow-up) and Model for End-stage Liver Disease (MELD) and Child-Pugh scores were calculated.

### Definitions, time points and groups

Patient data were assessed at baseline and at last follow-up (as defined by the last available imaging). For analysis, patients were devided into groups two times: (1) patients receiving early anticoagulation vs. patients not receiving anticoagulation/delayed start of anticoagulation and (2) patients receiving long-term anticoagulation vs. no anticoagulation/short-term anticoagulation. Long-term anticoagulation was defined as at least 9 months and until the end of follow-up. Major bleeding was defined according to the recent Baveno V consensus workshop [18]: active bleeding at endoscopy after the start of a specific drug treatment, with a drop of ≥3 g/dl of hemoglobin or bleeding requiring blood transfusion with an adjusted blood transfusion requirement index (ABRI) ≥0.75 at any time point.

### Statistics

All statistical analyses were performed using IBM SPSS Statistics 23 (SPSS, Armonk, NY, USA) and/or GraphPad Prism (version 6.00, GraphPad Software, La Jolla, CA, USA). Continuous variables were reported as mean ± standard deviation or median (interquartile range). Categorical variables were reported as number (proportion) of patients with/without the certain characteristic. The clinical course of PVT was described by calculating the proportion of patients that showed (i) regression, (ii) stable PVT and (iii) progression of PVT. We used the parametric Student’s t‑test for group comparisons if applicable. Otherwise, non-parametric tests were used (e. g. Wilcoxon Mann-Whitney U-test). Group comparisons of categorical variables were performed using either Pearson’s χ^2^-test or Fisher’s exact test. *P*-values <0.05 were considered statistically significant.

## Results

### Patients’ characteristics

**Fig. 1:**
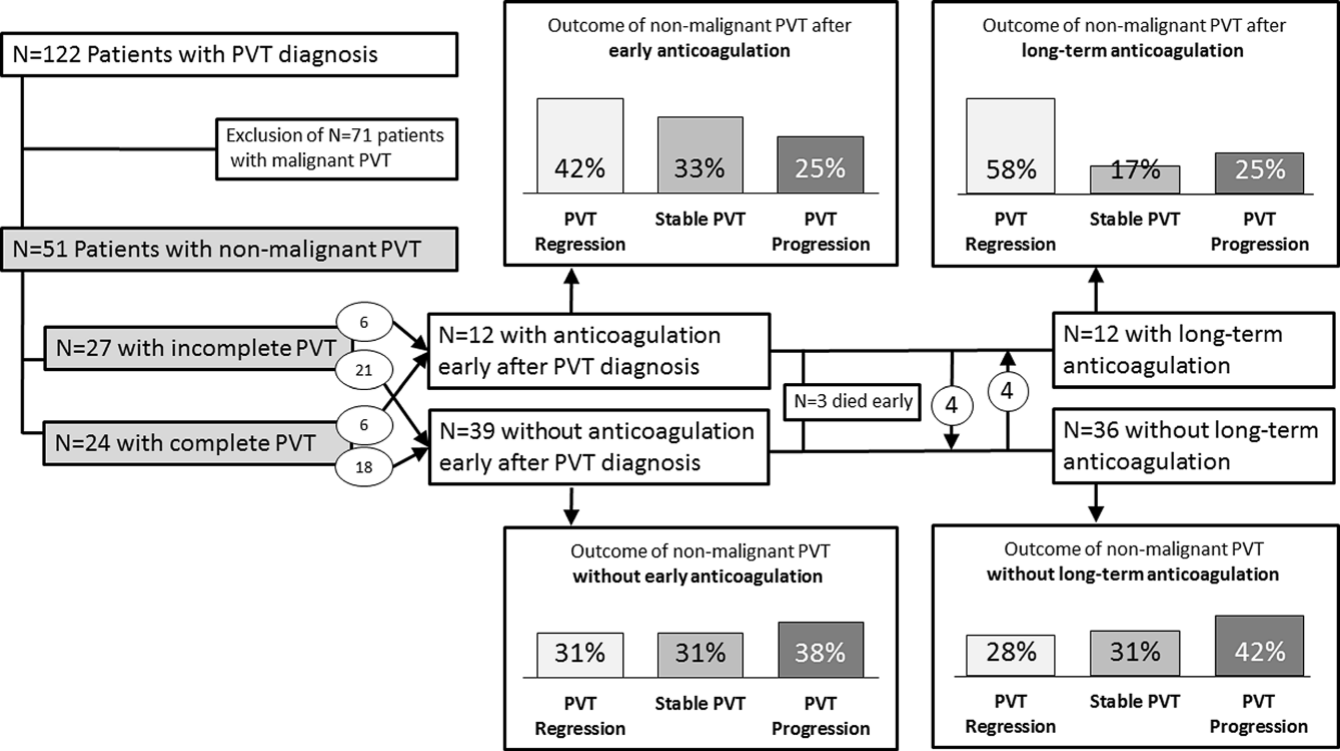
Consort flowchart and rates of PVT resolution, stabilization and progression

We identified a total of 122 patients diagnosed with PVT at our center, whereby 71 patients had to be excluded due to underlying malignancies. Thus, 51 patients with non-malignant PVT were included in this analysis (Fig. 1). The majority of patients were male (62.7%) with a mean age of 52.9 ± 12.5 years. The main etiology of liver disease was alcohol abuse in 24 patients (47.1%). Non-selective beta-blockers (NSBB) were used by 22 patients (43.1%) and most were on proton pump inhibitor (PPI) therapy (*n* = 45, 88.2%). Patients were followed for a median time of 44.1 (14.0–79.1) months. Anticoagulation treatment (*n* = 16 patients) was maintained for a median time of 12.0 (8.7–29.0) months (Table 1).

**Table 1:** Patient characteristics of patients with non-malignant PVT (*n* (%); mean ± SD; median (IQR))

Patients: *n* = 51 (100%)			
*Age (years)*	52.9 ± 12.5	*NSBB*	22 (43.1%)
*Gender (M/F, %M)*	32/19 (62.7%)	*PPI*	45 (88.2%)
*Etiology of liver disease*		*Ascites*	34 (66.7%)
Alcohol	24 (47.1%)	*Overt HE*	7 (13.7%)
Viral	6 (11.8%)	*Bilirubin (mg/dl)*	1.78 (1.88)
Other	21 (41.1%)	*Albumin (g/dl)*	33.3 ± 7.5
*Child-Pugh A*	14 (27.5%)	*INR*	1.45 ± 0.32
*Child-Pugh B*	19 (37.3%)	*CRP (mg/dl)*	1.26 (2.99)
*Child-Pugh C*	18 (35.2%)	*Platelets (G/l)*	113 (175)
*Child-Pugh score*	8.6 ± 2.7	*Severity of PVT*	
*MELD*	13.6 ± 6.5	Partial PVT	27 (52.9%)
*Diabetes*	5 (9.8%)	Complete PVT	24 (47.1%)
*Arterial hypertension*	6 (11.8%)	*Follow-up (months)*	44.1 (14.0–79.1)
*Varices*	39 (76.5%)	*Duration of anticoagulation (months) in n* *=* *16 patients*	12.0 (8.7–29.0)
*Previous variceal bleeding*	21 (41.2%)		

*CRP* C-reactive protein, *PVT* portal vein thrombosis, *MELD* Model for End-stage Liver Disease, *NSBB* non-selective betablocker, *IQR* interquartile range, *PPI* proton-pump inhibitor, *HE* hepatic encephalopathy, *INR* international normalized ratio

### Clinical presentation at PVT diagnosis

At PVT diagnosis, 14 patients (27.5%) had compensated cirrhosis (Child-Pugh stage A), while 37 patients had decompensated cirrhosis (Child-Pugh stage B: *n* = 19, 37.3%; Child-Pugh stage C: *n* = 18, 35.3%). The mean MELD score was 13.6 ± 6.5 points. Most patients had esophageal varices (*n* = 39, 76.5%) and 21 patients (41.2%) presented with a history of variceal bleeding. Two thirds of patients (*n* = 34, 66.7%) showed ascites at the time of PVT diagnosis, while overt hepatic encephalopathy was less common (*n* = 7, 13.7%). Half of patients (*n* = 27, 52.9%) showed incomplete PVT and *n* = 24 patients (47.1%) had complete PVT (Table 1).

### Early anticoagulation and regression rates of PVT

Following endoscopic evaluation, *n* = 12 patients (23.5%) were started with early (<10 days after PVT diagnosis) anticoagulation (Fig. 1). This decision to start early anticoagulation was at the discretion of the treating physician. In the group of patients being started with anticoagulation the proportion of female patients was higher (*p* = 0.04) and more patients without anticoagulation were taking NSBB (*p* = 0.03). The other baseline characteristics of patients receiving vs. not receiving early anticoagulation did not differ. Anticoagulation was initially achieved with LMWH and followed by oral anticoagulation with phenprocoumon (target INR levels 1.5–2.0).

When comparing PVT regression rates of patients with (*n* = 12) and without (*n* = 39) early AC, we found higher PVT regression rates (42% vs. 31%) and a lower rate of PVT progression (25% vs. 38%); however this result did not attain statistical significance (*p* = 0.67).

### Long-term anticoagulation and outcome of PVT

While three patients (5.9%) died early during follow-up and were excluded from further analysis, in four patients (8.3%) anticoagulation was stopped and in four other patients anticoagulation was started during follow-up. Finally, 12 patients (25.0%) were kept on long-term anticoagulation therapy (AC, Fig. 1). Patients receiving long-term AC showed a trend towards higher PVT regression rates (58% vs. 28% in patients receiving vs. not receiving long-term AC; *p* = 0.08).

### Long-term anticoagulation and outcome of PVT in patients with decompensated cirrhosis

**Table 2:** Development of portal vein thrombosis in patients with decompensated cirrhosis with vs. without long-term anticoagulation (*n* (%))

	No long-term anticoagulation (*n* = 29)	Long-term anticoagulation (*n* = 10)	*p*-value
Regression/resolution of PVT	7 (24.1)	7 (70.0)	*p* = 0.031
Stabilization of PVT	11 (37.9)	1 (10.0)	
Progression of PVT	11 (37.9)	2 (20.0)	

In total, 39 patients experienced decompensation of liver disease (history of or current ascites, hepatic encephalopathy or variceal bleeding) prior to PVT diagnosis. In this subgroup, a quarter of patients (*n* = 10, 25.6%) received long-term anticoagulation which resulted in a significantly higher rate of PVT regression when compared to patients without long-term anticoagulation (70% vs. 24%, *p* = 0.031; Table 2).

### Effects of anticoagulation on serum transaminases

**Fig. 2:**
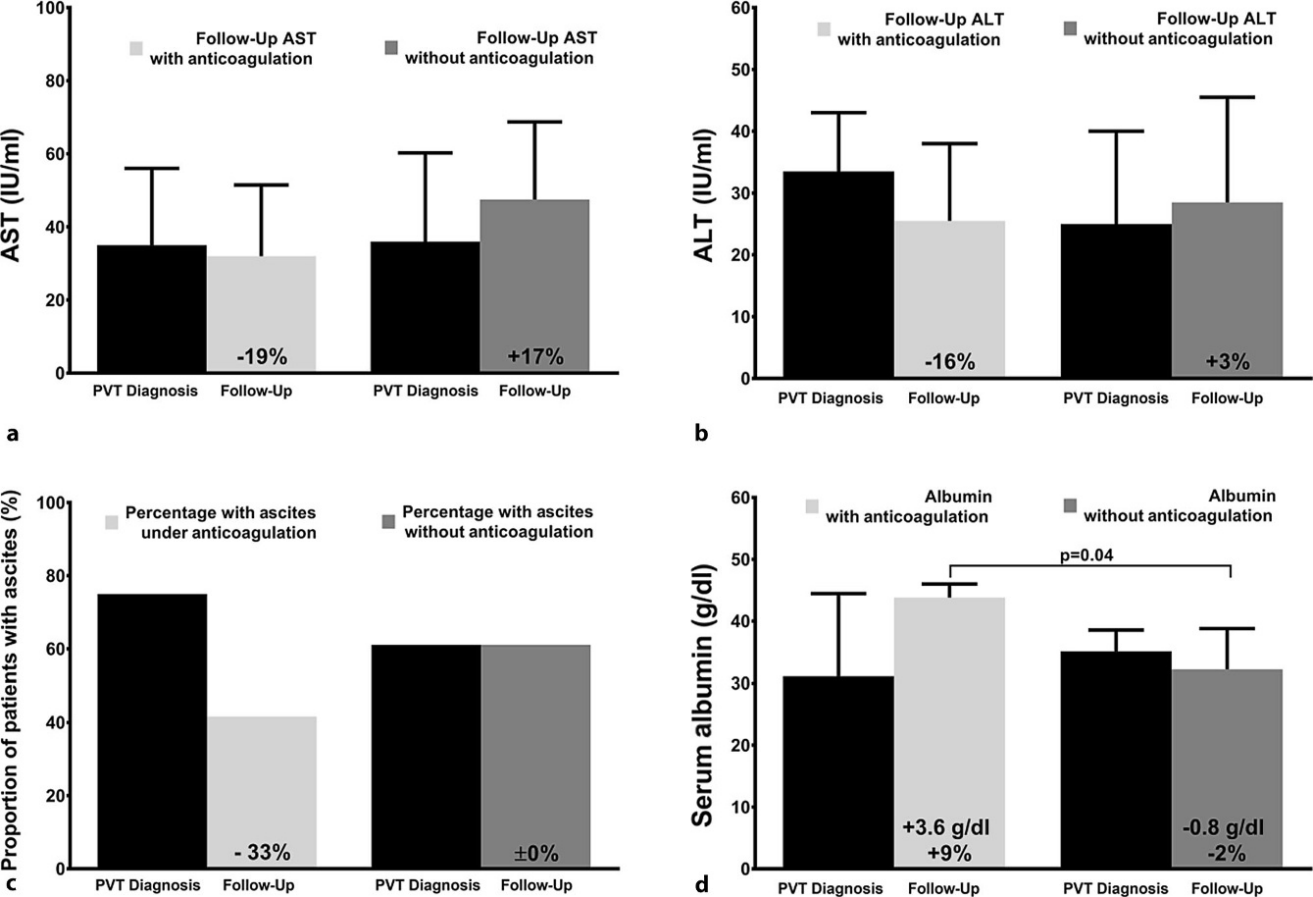
Course of nonmalignant PVT in patients with and without long-term anticoagulation. (**a**, **b**) Levels of serum aminotransferases at PVT diagnosis and during follow-up are shown separately for the groups of patients with long-term anticoagulation versus without long-term anticoagulation. While both aspartate aminotransferase (AST) and alanine aminotransferase (ALT) tended to decrease with long-term anticoagulation (−19%, −16%), AST/ALT increased in patients without anticoagulation (+17%, +3%). **c** While the proportion of patients with ascites decreased with long-term anticoagulation (from 9 to 5 patients, −33%), the number of patients with ascites remained unchanged without anticoagulation. **d** Serum albumin levels increased with long-term anticoagulation (+3.6 g/dl, +9%) but remained stable in patients without anticoagulation (−0.8 g/dl, −2%)

At PVT diagnosis, median levels of AST (35 [IQR:33] vs. 34 [IQR:36] IU/ml; *p* = 0.74) and ALT (34 [IQR:25] vs. 24 [IQR:23] IU/ml; *p* = 0.33) were similar in patients who subsequently received and in those who did not receive long-term AC (Fig. 2a, b). During long-term AC, liver enzymes decreased to median values of AST: 32 (IQR:24) IU/ml (−19%) and ALT: 26 (IQR:19) IU/ml (−16%) but increased without AC to AST: 47 (IQR:41) IU/ml (+17%) and ALT: 29 (IQR:25) IU/ml (+3%). However, the differences in the change of AST/ALT between patients with versus without long-term AC did not attain significance (AST: *p* = 0.43; ALT: *p* = 0.15).

### Impact of anticoagulation on ascites control and liver synthetic function

Interestingly, in four out of nine patients who had ascites at time of PVT diagnosis, ascites control was achieved after long-term AC. In contrast, in the group of patients without long-term AC, the number of patients with ascites remained unchanged (*p* = 0.24; Fig. 2c).

When assessing album levels as a marker of liver synthesis, there was no difference in albumin levels between patients with and without subsequent AC: 31.1 (IQR:13.6) vs. 35.1 (IQR:8.7) g/dl (*p* = 0.79) at the time of PVT diagnosis. However, after long-term AC, serum albumin levels increased to 43.8 (IQR:14.5) g/dl, while albumin levels remained unchanged in patients without AC during follow-up: 32.2 (IQR:10.5) g/dl. Importantly, albumin levels significantly improved in patients receiving long-term AC when compared to patients without long-term AC (+9% or +3.6 g/dl vs. −2% or −0.8 g/dl; *p* = 0.04; Fig. 2d).

### Safety of anticoagulation in patients with non-malignant PVT

**Table 3:** Bleeding events and sites during follow-up (*n* (%))

	Anticoagulation (*n* = 16)	No anticoagulation (*n* = 35)	*p*-value
*Bleeding episodes*	0	16	–
*Patients experiencing bleeding*	0 (−)	8 (22.9%)	0.045
*Bleeding sites*			
Gastric varices		3 (19%)	–
Esophageal varices		6 (38%)	
Gastric angiodysplasia		1 (6%)	
Esophageal ulcus post EBL		2 (13%)	
Portal hypertensive gastropathy		2 (12%)	
Duodenal ulcus		2 (12%)	

At baseline, 20 patients reported a history of variceal bleeding, 4 patients (33.3%) in the long-term AC group and 16 patients (44.4%) in the without long-term AC group (*p* = 0.50). In total, 16 bleeding episodes were registered in 8 patients (15.7%) within the study period and 2 patients (2.4%) died because of variceal bleeding (Table 3). Importantly, no bleeding episodes occurred in patients receiving AC (*p* = 0.045).

### DOAC treatment for splanchnic vein thrombosis

**Table 4:** Patient characteristics and outcomes of patients with splanchnic vein thrombosis treated with direct oral anticoagulants (DOACs; *n* (%); mean ± SD; median (IQR))

Patients: *n* = 10 (100%)			
*Age (years)*	50 ± 18	*Previous variceal bleeding*	2 (20%)
*Gender (M/F, %M)*	2/8 (20%)	*Bilirubin (mg/dl)*	0.49 (0.31)
*Liver cirrhosis*	3 (30%)	*Albumin (g/dl)*	39.1 ± 5.0
*Occlusion (partial/complete, % complete)*	4/6 (60%)	*INR*	1.21 ± 0.30
*Extension*		*DOAC used*	
*V. portae*	4 (40%)	Edoxaban (30 or 60 mg once daily)	4 (40%)
*V. portae* *+* *V. mes. sup*	2 (20%)	Apixaban (5 mg twice daily)	3 (30%)
*V. portae* *+* *V. mes. sup. + V. lienalis*	3 (30%)	Rivaroxaban (10 mg once daily)	2 (20%)
*V. lienalis*	1 (10%)	Dabigatran (110 mg twice daily)	1 (10%)
*Varices at treatment start*	8 (80%)	*Follow-up on DOAC treatment (months)*	9.2 (5.4–13.7)
*Outcome*			
Regression/resolution of PVT			2 (20%)
Stable PVT including patients with unchanged cavernous transformation of portal vein			8 (80%)
Bleeding events during DOAC treatment			1 (10%)

*V. mes. sup*. vena mesenterica superior, *INR* International Normalized Ratio

Additionally, 10 patients treated with DOACs for splanchnic vein thrombosis are reported separately (Table 4). The majority of patients were female (80%) and had a non-cirrhotic splanchnic vein thrombosis (70%). More detailed baseline characteristics can be found in Table 4. While 4 patients (40%) were treated with edoxaban (3 patients: 30 mg once daily; 1 patient: 60 mg once daily), 3 patients (30%) received apixaban (5 mg twice daily), 2 patients (20%) rivaroxaban (10 mg once daily) and 1 patient (10%) dabigatran (110 mg twice daily). The majority of patients were switched to DOACs in the setting of chronic PVT with cavernous transformation in order to prevent progression/extension of splanchnic vein thrombosis and potentially to improve splanchnic, intestinal and hepatic microcirculation. In this setting AC therapy can of course not recanalize PVT and treatment response can only be measured as non-progression of PVT. Therefore, comparison of treatment success between DOAC patients and patients treated with conventional AC was not performed. Median follow-up on DOAC treatment was 9.2 (IQR: 5.4–13-7) months. One bleeding event (i.e. bleeding from portal hypertensive gastropathy) occurred during DOAC treatment.

### Review of the literature on the course of PVT and efficacy of anticoagulation

**Table 5:** Studies reporting the course of cirrhotic PVT and efficacy/safety of anticoagulation

Study	Study design	Patients with PVT	Child A/B/C	Partial/complete	Anticoagulation given	Follow-Up	Improvement/Recanalization	Bleeding	Conclusion
Francoz C, Gut 2005 [11]	Prospective cohort study	29/251	4/19/6	20 (69.0%)/ 9 (31.0%)	19 (65.5%) received AC ([LMWH:nadroparin 5700 U/day]/VKA: INR 2–3) 10 (34.5%) did not receive AC	12.1 m	8/19 (42.1%) 0/10 (0%)	1 (bleeding from post-ligation ulcer)	PVT screening on OLT list AC is safe and impacts recanalization
Luca A, Radiology 2012 [8]	Prospective cohort study	42/42	Mean CPS: 8.1	42 (100%)/ 0 (−)	0 (−) received AC / 42 (100%) did not receive AC	27 m	(−) 19 (45%)	*n*/r	Partial PVT improved spontaneously in 45% Progression was not associated with outcome
Nery F, Hepatology 2015 [9]	Prospective cohort study	118/1243	863/380/0	101 (85.6%)/ 17 (14.4%)	6 (5.1%) received AC (*n*/r which anticoagulation)/ 112 (94.9%) did not receive AC	47 m	*n*/r	*n*/r	PVT development is associated with liver disease severity PVT does not lead to further progression of liver disease
Senzolo M, Liver Int 2012 [10]	Prospective cohort study	56/56	Treated: 11/16/8 Control: 5/9/7	24 (68.6%)/ 11 (31.4%)	33 (58.9%) received AC (LMWH:nadroparin 95 U anti-Xa/kg/day) 21 (37.5%) did not receive AC	21.6 m	21/33 (63.6%) 1/21 (4.8%)	3 (epistaxis, haematuria, ICB)	Anticoagulation (and TIPS) achieves good chance of repermeation and decreases PHT-related complications and progression
Amitrano, J Clin GE 2010 [12]	Prospective cohort study	39/39	B/C: 46.4%	23 (82.1%)/ 5 (17.9%)	28 (71.8%) received AC (LMWH: enoxaparin 200 U/kg/day) 11 did not receive AC	11 m	21/28 (75%) *n*/r	2 (Mild PHG-related anemia)	LMWH is safe and effective for cirrhotic patients with PVT
Delgado MG, CGH 2012 [13]	Retrospective cohort study	55/55	25/21/9	41 (74.5%)/ 14 (25.5%)	55 (100%) received AC (LMWH/VKA)	19 m	33/55 (60%) Rethrombosis in 21 (38.5%)	11 (variceal bleeding, GI-bleeding, others)	AC is safe with recanalization rates of 60% and should be maintained to prevent Re-PVT
Werner KT, DigDisSci 2013 [14]	Retrospective cohort study	69/537	*n*/r MELD: 7–29	*n*/r	28 (40.6%) received AC (warfarin: INR: 2–3)/ 41 did not receive AC	10.1 m	23/28 (82%)	1 (vaginal)	AC is safe and effective in patients with ESLD awaiting OLT
Cui SB, Eur J GH 2015 [19]	Prospective randomized cohort study	65/65	CPS: 7.0	54 (83.1%)/ 11 (16.9%)	65 (100%) received AC (LMWH: Enoxaparin) *n* = 34 1.5 mg/kg (q24h) *n* = 31 1 mg/kg (q12h)	6 m	51/65 (78.5%)	No VB 10 mild bleeding	Enoxaparin is effective/safe, 1 mg/q12h seems better than 1.5 mg/q24h for cirrhotic PVT

*AC* anticoagulation, *CPS* Child-Pugh score A/B/C, *HBV* hepatitis B virus, *INR* international normalized ratio, *LMWH* low-molecular weight heparin, *n/r* not reported, *OLT* orthotopic liver transplantation, *PVT* portal vein thrombosis, *VKA* vitamin K antagonists, *PHT* portal hypertension, *MELD* Model for End-stage liver disease, *ESLD* End-stage liver disease, *q24h* every 24 hours, *q12h* every 12 hours

Of the studies eight (five prospective cohort studies, one prospective randomized cohort study and two retrospective cohort studies) were identified that reported the outcome of PVT and data on the efficacy and safety of anticoagulation therapy in cirrhotic patients with non-malignant PVT [8–14, 19] (Table 5). Most studies used either LMWH (mostly nadroparin or enoxaparin) or VKA (mostly after starting AC with LMWH first). The reported PVT resolution rates ranged from 42.1% to 82% and were consistantly higher with anticoagulation [11, 14]. The incidence of bleeding was low and the severity of the bleeding events reported was mild or moderate [10, 12, 14, 19]. A VKA therapy was usually adapted to reach an INR between 2–3 [11, 14].

## Discussion

In our series of patients with non-malignant PVT, anticoagulation resulted in a trend towards higher regression rates and a decreased risk of PVT progression. While the risk of PVT progression could not be completely abolished by the use of anticoagulation, we observed beneficial impacts on hepatic inflammation and liver function: Interestingly, in patients with long-term anticoagulation, the levels of transaminases (AST/ALT) decreased numerically and albumin as a parameter of liver synthesis function increased significantly. Furthermore, in a subgroup analysis including patients with previously decompensated liver disease, we observed statistically significant higher PVT regression rates with long-term anticoagulation. This is especially important as patients with decompensated cirrhosis are at increased risk of developing complications and there is a pronounced need for successful treatment of PVT [20–22]. Importantly, the incidence of gastrointestinal bleeding was not increased with anticoagulation and even in patients presenting with previous variceal bleeding at PVT diagnosis no re-bleeding occurred during follow-up. It can be concluded that AC in cirrhotic patients even with previous variceal bleeding can be started safely after endoscopic exclusion of potential high-risk bleeding sites. In our clinic, if high-risk varices are present at screening, anticoagulation therapy is started after appropriate treatment of varices (verified hemodynamic response to NSBB therapy or endoscopic band ligation).

The observed PVT regression rate in our patient series was 58% when anticoagulation was given as long-term treatment. This finding is consistent with previous studies reporting regression/resolution rates ranging from 42.1% [11] up to 82% [14]. While the proportion of patients achieving regression of PVT on anticoagulation was numerically higher in the whole cohort and statistically significantly higher in patients with decompensated liver disease, the low number of patients with compensated liver disease treated with anticoagulation (*n* = 2) limited the statistical power of our analysis in the whole cohort, and thus, the difference in resolution/recanalization rate did not attain statistical significance. Nevertheless, the numerically higher PVT regression/resolution rates with anticoagulation represent a clinically relevant effect size and anticoagulation should be offered to all patients with PVT in the absence of absolute contraindications (such as active gastrointestinal bleeding or a history of intracranial bleeding).

While bleeding rates and severe bleeding events were not consistently reported across all studies, bleedings were usually mild and variceal bleeding was rare [10, 11, 13, 14]. In our study, no bleeding occurred in patients receiving conventional AC. In patients treated with DOACs one bleeding episode occurred; however, bleeding was controlled endoscopically (with argon plasma coagulation) and treatment could be restarted. This is in line with the results of another study, in which the authors did not observe an increased bleeding risk in patients with cirrhosis receiving novel direct oral anticoagulants (rivaroxaban or apixaban), when compared to LMWH/phenprocoumon [23].

The safety of anticoagulation in patients with PVT could also be explained by potential beneficial effects on intrahepatic vascular resistance, which were recently described in an experimental study in cirrhotic rats treated with enoxaparin [16]. Interestingly, long-term administration of enoxaparin reduced portal pressure by inhibiting hepatic stellate cell activation (dynamic component of portal hypertension), decreasing liver fibrosis (structural component of portal hypertension) and by prevention of intrasinusoidal microthrombosis [16].

We found a decrease in serum transaminases with long-term anticoagulation, as both AST and ALT levels dropped (by −19% and −16%, respectively) while AST/ALT increased without long-term anticoagulation (+17%/+3%). This suggests that the beneficial effects of enoxaparin described in the experimental study by Cerini et al. [16] might also lead to a decrease in hepatic necroinflammation in cirrhotic patients with PVT. Most interestingly, long-term anticoagulation was also associated with a better control of ascites during follow-up, as four out of nine patients with ascites at PVT diagnosis achieved control of ascites, while the number of patients with ascites remained stable in patients without long-term AC. In addition, we also found a significant increase in serum albumin under long-term anticoagulation (+3.6 g/dl /+9%) while serum albumin levels were almost unchanged in patients without long-term anticoagulation.

Our findings indicate that anticoagulation therapy leads to improvements in liver dysfunction and is in line with a previous study showing a decrease in hepatic decompensation with LMWH [17]. In line with our subgroup analysis, in the latter study, all patients were already decompensated at start of LMWH (Child-Pugh B7-C10 was an inclusion criterion) and no patients had PVT at inclusion. The study was designed to assess if prophylactic LMWH can prevent PVT, which was the case, but the even more interesting findings were the delay in hepatic decompensation during enoxaparin treatment (11.7% vs. 59.6% in the control group) and increased survival rate in the enoxaparin treated group [17].

While the study by Villa et al. [17] and our findings in this retrospective analysis would both suggest a clinical benefit of anticoagulation on liver dysfunction in cirrhosis, these effects have to be confirmed in larger prospective studies in patients with cirrhosis with and without PVT. The monitoring of traditional anticoagulation in patients with cirrhosis represents a clinical challenge [24, 25] and while DOACs seem to be attractive alternative options, their efficacy and safety in cirrhosis has yet to be evaluated [26].

In conclusion, our findings support the use of AC in patients with non-malignant PVT, since AC is not only safe and associated with superior PVT regression/resolution rates, but might also decrease hepatic inflammation and improve liver synthesis.
